# Research on Mechanical and Shrinkage Characteristics of a Resource-Based Cement Solid-Waste Concrete

**DOI:** 10.3390/ma17010177

**Published:** 2023-12-28

**Authors:** Shikai Ning, Xidong Jiang, Bin Li, Long Shan, Hongbo Li

**Affiliations:** 1 China Railway Construction Bridge Engineering Bureau Group Northwest Engineering Co., Ltd., Yinchuan 750021, China; xdongjiang@163.com (X.J.); libinztj@163.com (B.L.); 2 China Railway Construction Bridge Engineering Bureau Group, Yinchuan 750021, China; 3 College of Civil and Hydraulic Engineering, Ningxia University, Yinchuan 750021, China; 12022131152@stu.nxu.edu.cn

**Keywords:** concrete, environmental protection building materials, shrinkage power, interlocking cementation, dense association

## Abstract

Recycling of multi-source solid waste is of great benefit to energy conservation and environmental governance. In this paper, a new type of environmental protection concrete for railway accessory facilities was prepared from silicon-manganese slag, steel slag, fly ash and recycled macadam. Seven kinds of concrete with different mix proportions were designed. Through unconfined compressive strength, splitting, drying shrinkage and temperature shrinkage tests, the multivariate changing trends of steel slag content, cement dosage and age on the anti-interference ability of concrete were investigated. The main mechanisms of the development of mechanical and dry shrinkage properties were revealed by the hydration process of 3SR-60. The results show that 3SR-60 had better mechanical strength under the same cement dosage. The temperature shrinkage strain decreased and then increased with the rise of the proportion of waste residue, increased with the addition of cement dosage and decreased first and then increased with the descent in the temperature. The temperature shrinkage coefficient reached the lowest value at 0–10 °C. The drying shrinkage coefficient decreases with the increase in the proportion of waste residue and increases with the increase in cement dosage. The dry shrinkage strain increased rapidly during the first 8 days and became almost constant after 30 days. Cementation of calcium silicate hydrate (C-S-H) and ettringite (AFt) developed continuously and filled the internal pores of the structure, interlocking and cementing with each other, which made the microstructure develop from a three-dimensional network to a dense complex, and the macro dimension was reflected in the enhancement of the power to resist external interference. The conclusion of the test summarized that SR-60 had preferable mechanical and shrinkage performance.

## 1. Introduction

Industrial solid waste refers to the waste residue, dust and other wastes in the chemical industry, electric power and metal smelting [1,2,3]. In recent years, the annual output of industrial waste residue in China has been as high as 3.6 billion tons [4]. According to reference [5], steel slag is a by-product of crude steel smelting, and its output can reach about 12% of crude steel output. In addition, the crude steel output of China in 2022 was 1.018 billion tons, ranking first in the world, which indicates that the steel slag output reached 122 million tons in 2022. The utilization rate of steel slag is less than 30% because it is mostly stored and disposed [6]. Related to this, it is noteworthy that forging 1 t manganese will cause the accumulation of 10 t silicomanganese slag [7], and fly ash belongs to the main waste of coal-fired power plants, which can produce 0.7 × 10^9^ t fly ash per trillion kW·h of power supply [8]. It is estimated that the output of fly ash in China will reach 854 million tons in 2023, posing a serious threat to green ecology [9,10]. With the vigorous development of urbanization in Ningxia, the output of building rubble is increasing year by year, but the utilization rate is difficult to meet the standard, which seriously harms the water and soil environment [11,12,13,14]. Therefore, seeking the harmless disposal of solid waste has become an important direction to relieve the shortage of resources and the sustainable development of environmental protection.

Nowadays, a large number of academic talents have carried out relevant research on the utilization of multi-source solid waste to manufacture railway maintenance building materials, and scientifically predicted the resistance to external interference of concrete. The gel obtained by hydration of free calcium oxide (f-CaO) and magnesium oxide (f-MgO) in silicomanganese slag and steel slag has the characteristics of micro-expansion [15,16], so it can compensate for the shrinkage defect of concrete. At the same time, this makes the whole concrete frame denser to a certain extent and improves its mechanical properties after mixing. Gao et al. [17], based on the characteristic that the appearance texture and density of steel slag is denser than natural crushed stone, found that the shrinkage and the mechanical performances became stronger after mixing steel slag into concrete. Pasetto et al. [18] optimized the ratio of steel slag and crushed stone aggregate to modify the mechanical property and durability of the mixture. Miah et al. [19] found that steel slag can form an alkaline medium after depolymerization, which stimulated the low-activity silicic acid substances in the system to generate additional C-S-H, and the overall mechanical properties and durability were optimized. Tamayo et al. [20] found that building materials containing silicomanganese slag will weaken some mechanical properties, but they were suitable for non-structural concrete. Frías et al. [21,22] summarized that the strength in 7 days of the colloidal sand was weaker than that of the control group, but the later strength was gradually the same. At the same time, silica manganese slag can elevate the corrosion resistance and durability of cement paste in NaCl and Na_2_SO_4_ media. Tong et al. [23] investigated the drying shrinkage characteristics of steel slag and crushed stone stabilized by fly ash and cement, and the results manifested that the construction temperature of the mixture should be controlled between 5–25 °C. Fly ash is widely used in construction fields such as goaf [24,25]. Zhang et al. [26] concluded that fly ash can enhance the working performance of binders and present important economic and environmental benefits. Gravel can be used for slope preparation after crushing and screening, which is in line with the national green development trend. Li et al. [27] found that gravel weakened the application efficiency of the mixture, but the addition of 4% cement dosage met the requirements of the specification. Yan et al. [28] evaluated that when the content of rice husk ash (RHA) in macadam stabilized by RHA was controlled within 40%, the combination property of the mixture is better. Xue et al. [29] discovered that the mechanical properties of the mixture in the same medium were positively correlated with the proportion of crushed stone.

In recent years, prominent representatives in the field of concrete have proposed methods for testing concrete performance. This provides a rich theoretical guidance for the sustainable utilization of concrete. Aggelis et al. [30] studied the response pattern between the fracture mode and the service life of concrete. The results show that the combination of acoustic emission features is helpful to characterize the transition between major fracture modes of concrete and to monitor the structural health of concrete. This provides a theoretical guide for field application. Naoum et al. [31] introduced a novel structural health monitoring (SHM) system. The experimental results show that the SHM system can be used to rapidly assess the damage level in the early seismic loading stage of existing reinforced concrete buildings.

**Figure 1:**
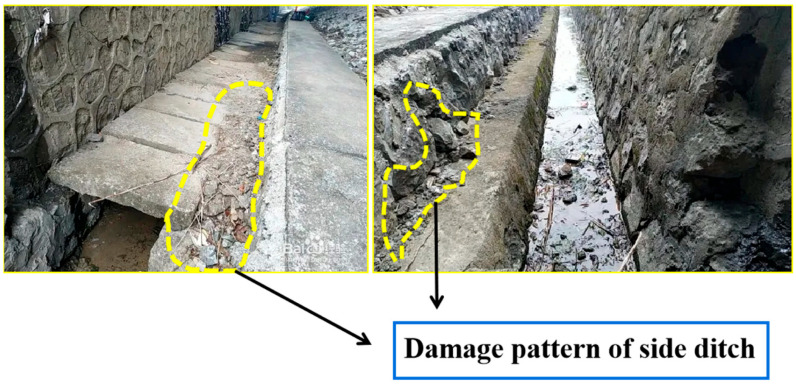
Failure mode of drainage ditch.

These outstanding scientific researchers have provided theoretical support and technical guidance for the comprehensive utilization of multi-source solid waste and promoted the development trend of low-carbon building materials to some extent. However, the research on using industrial waste residue and construction waste as raw materials for railway drainage ditches is still very limited. With the continuous expansion of the railway scale in China, the related maintenance construction has entered a golden stage. Railway construction is often carried out in the open air, and different regions will cause geological and ecological differences. Especially in mountains and hills, the terrain fluctuates greatly. Taking Yinba Railway in China under construction as an example, the cold winter of Ningxia in China and the large temperature difference between day and night make the risk of strength damage and cracking higher. In addition, cracks occur in winter, and the accumulated water is easy to form frost heaving after soaking in cracks, which deepens the severity level of side ditches. Concrete cracking is closely related to its shrinkage degradation, which is caused by the volume deformation created by the shrinkage of the mixture in different humidities and temperatures. This kind of volume deformation gradually extends to form reflection cracks, which not only shortens the service life of the drainage ditch, but also increases the construction and maintenance cost. The failure modes of other railway drainage ditches is displayed in Figure 1.

The strength and shrinkage performance of concrete directly affects its service life, so it is particularly necessary to comprehensively investigate the mechanics and contractile properties of building materials to ensure the safe use of railway series construction. Therefore, this study selected cement and fly ash as cementing materials, steel slag, silicon-manganese slag and recycled macadam as composite aggregates. Furthermore, it explores the development trend of the strength and shrinkage characteristics of the aforementioned materials under the influence of waste slag ratio, cement dosage and time, and clarified the microstructure of the concrete by Scanning Electron Microscope (SEM). Then, it comprehensively investigated the formation mechanisms of strength and shrinkage performance, aiming at providing a reference for relieving the pressure of natural sand and gravel mining, consuming industrial solid waste, building a sustainable green economic system in China and promoting the early formation of social double carbon strategy [32,33].

## 2. Materials and Methods

### 2.1. Materials

The steel slag came from the Ningdong waste dump in Ningxia, silicon-manganese slag came from a waste iron and steel plant in Ningxia, fly ash was obtained from a thermal power plant in Ningxia, cement was purchased from Yongan cement production base of Yongan Cement Co., LTD of Henan Province in China and the variety grade was ordinary Portland cement 42.5. Recycled macadam was sent to the experimental raw material storage bin after being crushed and screened by Waste Concrete Company of Ningxia in China. The chemical compositions of raw materials were detected by X-ray fluorescence instrument, and the results are shown in Table 1. The main chemical compositions of steel slag, silicon-manganese slag, fly ash and crushed stone are CaO, SiO_2_ and Al_2_O_3_, accounting for more than 60% by mass, with relatively stable chemical properties and potential pozzolanic and gelling activity [34].

**Figure 2:**
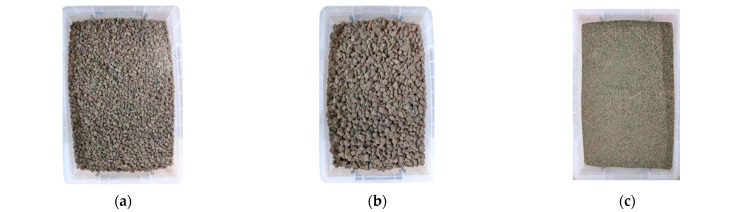

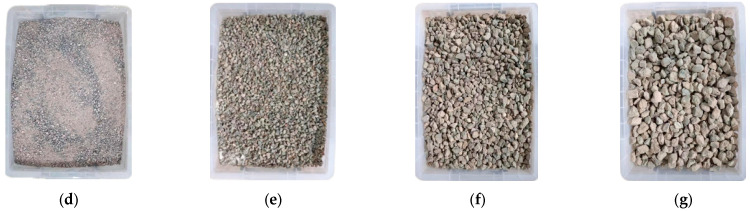
Appearance and morphology of aggregates: (**a**) 2.36–9.5 mm steel slag, (**b**) 9.5–16 mm steel slag, (**c**) 0–2.36 mm silicon-manganese slag, (**d**) 0–2.36 mm recycled macadam, (**e**) 2.36–9.5 mm recycled macadam, (**f**) 9.5–16 mm recycled macadam, and (**g**) 16–26.5 mm recycled macadam.

To fully reveal the change mechanism of the aggregate medium on concrete strength and shrinkage characteristics, the crushing value, water absorption and apparent density of 2.36–9.5 mm and 9.5–16 mm steel slag, 0–2.36 mm silicon-manganese slag, 0–2.36 mm, 2.36–9.5 mm, 9.5–16 mm and 16–26.5 mm recycled macadam were tested according to the corresponding provisions of JTG E42-2005 [35]. The results are shown in Table 2 and the appearance of aggregates are shown in Figure 2.

### 2.2. Schemes

#### 2.2.1. Distribution of Raw Materials in Specimens

According to the results of crushing and screening, it was found that 0–2.36 mm silicon-manganese slag accounted for more than 75% of the total silicon-manganese slag, and 2.36–16 mm steel slag accounted for more than 68% of the total steel slag. Combined with the prescribed range of cement and fly ash stabilization materials in JTG/T F20-2015 [36], and the grading specifications of CF-B-2S, the specimen of silicon-manganese slag with 0–2.36 mm and 19.2% content (steel slag content was 0), 2.36–9.5 mm and 9.5–16 mm recycled macadam of 0, 30%, 60% and 90% was replaced by steel slag with the corresponding particle size and 2%, 3% and 4% cement dosage (the steel slag replacement rate was 60%) were designed, respectively. The evolution rules of silicon-manganese, steel slag replacement rate and cement dosage on the strength and shrinkage performance of concrete were investigated in different environments. The mix ratios of concrete established in the test are shown in Table 3, where 3–0 indicates that the aggregate is only recycled macadam at 3% cement dose, 3SR-60 demonstrates that the cement dose in the specimen mixed with silicon-manganese slag is 3% and SR-60 introduces that the replacement rate of steel slag is 60%, and so on.

#### 2.2.2. Preparation and Curing of Concrete Specimens

**Figure 3:**
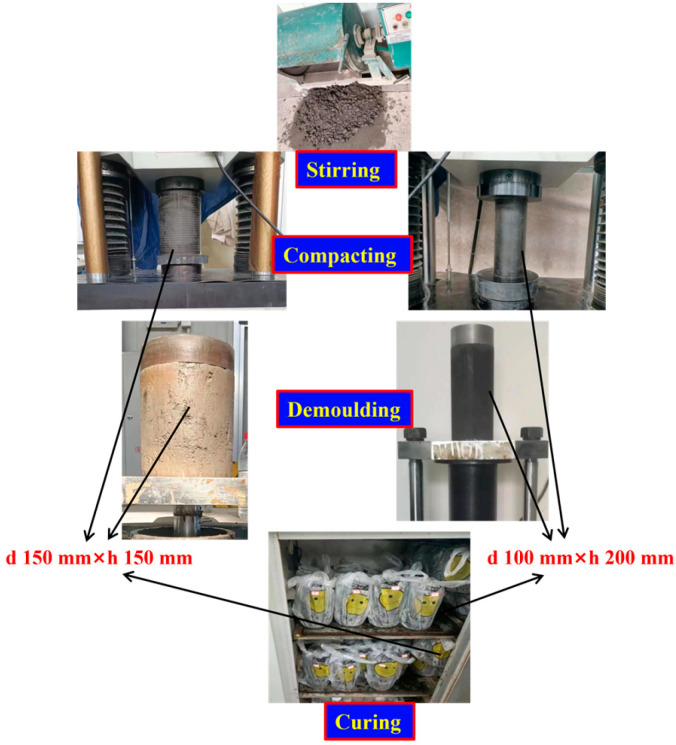
Preparation and curing of specimens.

According to the contents of T0804-1994, T0843-2009 and T0845-2009 in JTG E51-2009 [37], the compaction test to obtain the best moisture content of 7 types of concrete, the preparation and health preservation of concrete were carried out successively. The concrete specimens were formed by static pressing method. In addition, the specimens were demolded after the initial setting time of cement, and the compaction degree was 98%. Specimens were cured at a temperature of 20 ± 2 °C and humidity above 95% (standard curing method) for 10 d for unconfined compressive strength, splitting, dry shrinkage and temperature shrinkage tests, while specimens at 30 d and 90 d were only used for testing unconfined compressive strength and splitting strength. All specimens were immersed in water for 24 h on the last day of curing. The purpose is to investigate the water stability of concrete. A cylindrical specimen with a diameter of 150 mm and a height of 150 mm was selected for the mechanical properties test. Due to the limited scale of the test, a cylindrical specimen with a diameter of 100 mm and a height of 200 mm was used for the shrinkage test with a replacement beam shape. The preparation and curing processes of specimen is shown in Figure 3.

#### 2.2.3. Detection of Unconfined Compressive Strength and Splitting Strength

**Figure 4:**
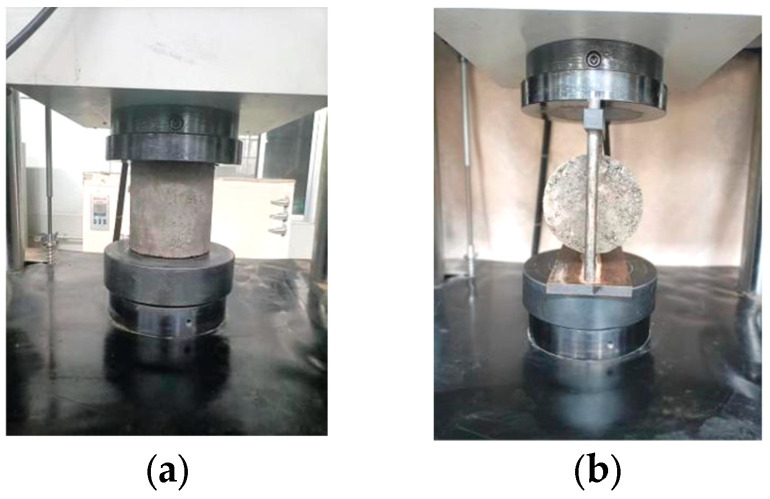
(**a**) The test of unconfined compressive strength and (**b**) the test of splitting strength.

According to T0805-1994 and T0806-1994 in JTG E51-2009 [37], the unconfined compressive strength and splitting strength of specimens at 10, 30 and 90 days were tested. During the test, 13 specimens were prepared for each mix ratio and each curing age. The whole process needed to be completed by microcomputer control of electro-hydraulic servo universal testing machine equipment. The pressure rate was 1 mm/min, and the maximum axial load was 1000 kN. The test of mechanical properties of specimens is shown in Figure 4.

#### 2.2.4. Detection of Temperature Shrinkage

**Figure 5:**
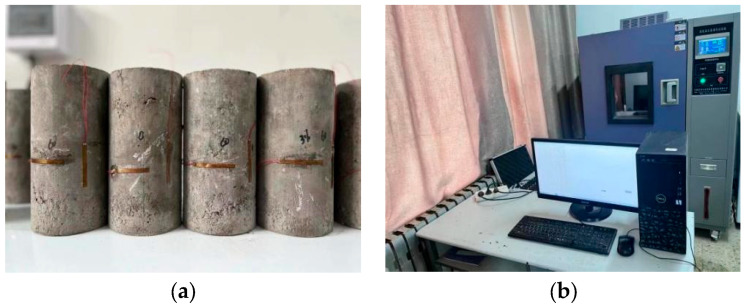
Temperature shrinkage test: (**a**) specimens with attached strain gauge and (**b**) overall schematic diagram.

Based on the scheme T0855-2009 in JTG E51-2009 [37], the strain gauge method was used to conduct a temperature shrinkage test to explore the evolution characteristics of the temperature shrinkage coefficient with the change in temperature specification. During the test, 6 specimens were prepared for each mix ratio: three for measure of the shrinkage deformation of the concrete, and three for measure of the dry shrinkage water loss rate of the concrete. Firstly, the soaked specimens were baked in the oven at 105 °C for 12 h to constant weight, and then put into the drying oven to normal temperature. The purpose is to avoid the influence of moisture on the temperature shrinkage test results. Then, sandpaper was used to sand the center of the side of the specimen and an electric blower was applied to remove the surface dust. Finally, the vertical strain gauge, circumferential strain gauge and temperature compensation plate were pasted, and the lead wire was connected to the strain gauge interface smoothly. The test temperature was set to range from 60 °C to −20 °C, from high temperature to low temperature; the decline rate was 0.5 °C min^−1^, the temperature interval of each stage was 10 °C and the heat preservation was conducted for 3 h. Three parallel tests were carried out for each group of matching test pieces, and the average value of the results was taken. The prepared samples were placed inside the test chamber for debugging high- and low-temperature alternating humidity and heat adjustments. After the contact ports are fully prepared, the test chamber program was opened, and the required data were recorded. The temperature shrinkage test is shown in Figure 5 and the temperature shrinkage coefficient is calculated by a mathematical Formula (1):

(1) $$\alpha_{t}=\frac{\varepsilon_{i}}{t_{i}-t_{i-1}}$$

where *α*^t^ is the temperature shrinkage coefficient (µε·°C^−1^), *t*_i_ is the i temperature interval (°C) and *ε*_i_ is the shrinkage strain at the i temperature.

#### 2.2.5. Detection of Dry Shrinkage Test

**Figure 6:**
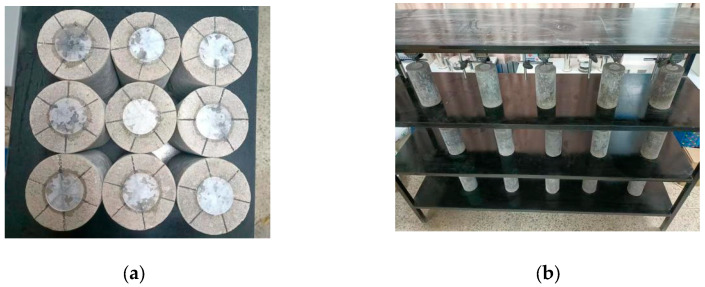
Dry shrinkage test: (**a**) specimens stuck with plexiglass sheet and (**b**) overall schematic diagram.

According to the procedure specified of T0854-2009 in JTG E51-2009 [37], the dry shrinkage characteristics of the specimens were investigated, and the formation trend of total water loss rate, total dry shrinkage strain and dry shrinkage coefficient with the dry shrinkage age were analyzed in turn. During the test, 3 specimens were prepared for each proportion of concrete to determine the shrinkage deformation of the concrete. Firstly, the surface water of the soaked specimen was removed with a dry cloth, and the surface of the specimen was polished and regularized by an angle grinder. The height and quality of the specimen were recorded. Next, epoxy resin was used to enhance the surface flatness of the specimen and the plexiglass sheet was glued in the center of the upper surface of the specimen. Finally, the specimen was smoothly placed on the upper layer of the drying table (the control temperature was 20 ± 1 °C, and the relative humidity is 60% ± 5%), and the dial gauge was fixed at the bottom of the drying table and made slightly contact with the glass sheet, and the initial data were recorded. Six samples were tested for each type of concrete, three for the detection of water loss rate and three for the measurement of dry shrinkage strain, and the average value of the results was taken. The dry shrinkage test is shown in Figure 6.

After the specimens were placed on the dry shrink table, the dial readings were observed and recorded every 1 day in the age range of 0–10 days. Dial readings were observed and recorded every 2 days for a period of 10 to 30 days. At 30–60 days, dial readings every 5 days were observed and recorded. In the age range of 60–90 days, the dial readings were read and recorded every 15 days. After the last dial reading within 90 days, all samples were placed in the oven to dry to a constant weight, and then the quality of the samples was tested and recorded. At the same time, during the reading process, all specimens need to be weighed individually.

In the unconfined compressive strength and splitting strength data, the final result was obtained based on the average of 13 data values. Among the 13 data values, the triple mean square error method was used to eliminate outliers, allowing for 2–3 outliers. If the abnormal value exceeded the above regulations, the test shall be repeated until the requirements were met. In the dry shrinkage and temperature shrinkage tests, the final result was the average of three parallel tests. If the range and average of these three values exceeded 30%, retest until the test results met the requirements.

#### 2.2.6. Analysis of Hydration Mechanism

**Figure 7:**
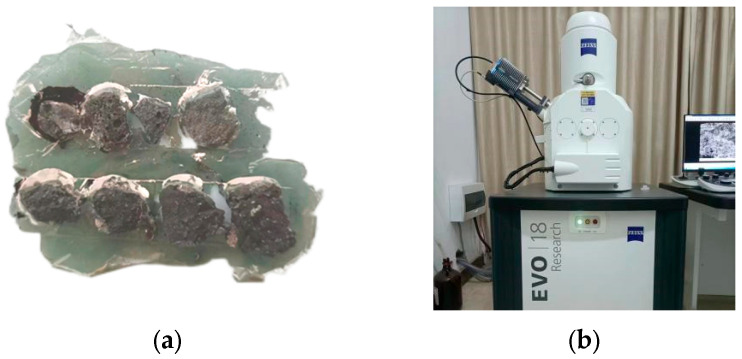
Analysis process of SEM: (**a**) sample preparation and (**b**) test equipment.

The micro changes of concrete can map the macroscopic special phenomenon, and discussing the evolution of the internal body structure of specimens can reveal the principle of the difference of strength and dry shrinkage characteristics of specimens in different periods. Based on the multiple extension background of steel slag, silicon-manganese slag, recycled macadam and time, the study further investigated the micro-morphology of concrete at different stages to find and investigate the main structures that affected the strength and dry shrinkage performance, which were conducive to the comprehensive study of its performance change mechanisms. The SEM instrument of model EVO 18 from Zeiss (Jena, Germany) was used as the micro test equipment, and the detection multiple was controlled at 5×–1,000,000×. The preparation and test equipment of samples are shown in Figure 7.

## 3. Result Analysis and Discussion

### 3.1. Detection of Optimal Moisture Content

Table 4 summarizes the test results of optimal water content and maximum dry density of 7 types of concrete, and it can be clearly observed that the optimal water content shows a decreasing trend with the increase in the steel slag mixing ratio. At a 3% cement dose, the optimal water content of the four steel slag specifications decreases by 0.47% on average, while the maximum dry density increases by 0.117 g/cm^3^ on average. This is because the steel slag is smoother and denser than the recycled crushed stone, which in turn showed the phenomenon that the optimal water content decreased, and the maximum dry density rose. The porous and soft properties of silicomanganese slag are opposite to those of steel slag. When the cement dosage increased from 2% to 4%, the optimal water content of the concrete SR-60 increased by 0.3% and 0.4%, respectively, due to the fact that the cement contains most of the more hydrophilic oxides, and the cement hydration produced more C-S-H and other cement-forming groups with better compactness, which facilitated the maximum dry density of the concrete.

### 3.2. Detection of Unconfined Compressive Strength

**Figure 8:**
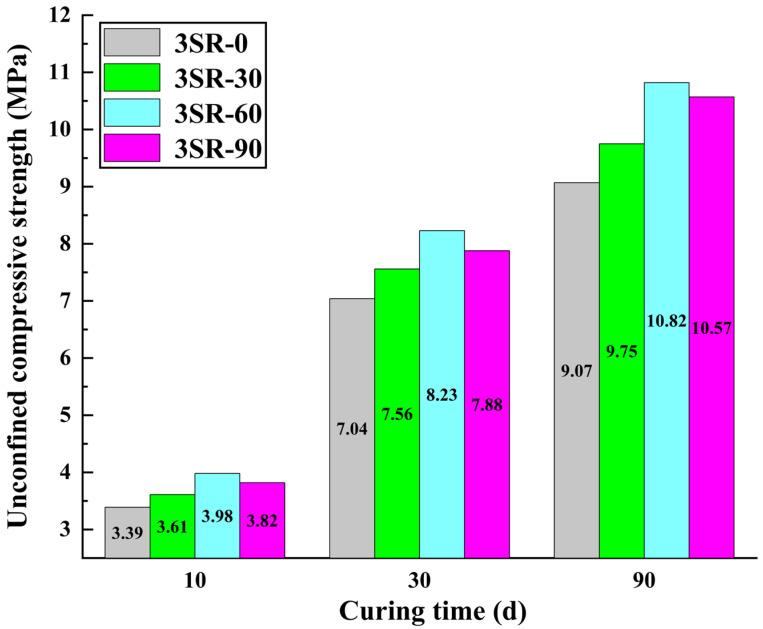
Test results of steel slag mixing ratio and unconfined compressive strength.

Figure 8 investigates the laws of unconfined compressive strength of concrete with 3% cement dosage under the influence of steel slag mixing ratio and curing time. It can be found that the compressive strength of concrete increases first and then decreases with the increase in steel slag content, and the strength of 3SR-60 reaches the peak of each age, demonstrating that a certain proportion of steel slag can optimize the compressive strength of concrete. However, the development of strength was inhibited by the addition of too much steel slag. This is because the smaller crushing value of steel slag made the concrete with the right amount of steel slag more resistant to external interference. Conversely, too much steel slag made the micro-expansion effect of concrete superimposed, resulting in the overall structure becoming soft, which was not conducive to the strength increase. The strength of 3SR-60 at 10, 30 days and 90 days was 3.93 MPa, 8.23 MPa and 10.82 MPa, respectively. Compared with the curing time of 10 days, the growth rate of 30 days and 90 days was 109.4% and 175.3%, and the growth rate degraded. The main mechanism of this difference was that active oxides such as CaO in waste slag and cement participated in the reaction in large quantities in the early stage, while the development of strength slowed down due to the reduction in the number of raw materials involved in the later stage reaction. The specimens of 3SR-0, 3SR-30 and 3SR-90 expressed similar test rules and differential mechanisms to 3SR-60, so it is not necessary to go into details here. In summary, the concrete with preferable mechanical properties is 3SR-60.

**Figure 9:**
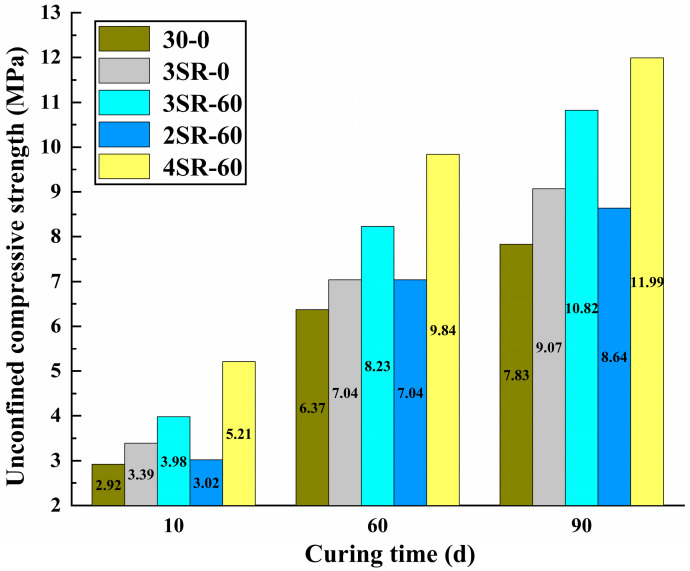
Test results of dosage of silicon manganese slag and cement and unconfined compressive strength.

Based on concrete 3SR-60 with better mechanical strength, the compressive strength laws of concrete 30-0, 3SR-0, 2SR-60, 3SR-60 and 4SR-60 at different ages were normalized to reveal the influence of dosage of silicon-manganese slag and cement on the strength of concrete. The results are shown in Figure 9. It can be found that the strength formation trend of the same concrete at different ages is similar, so the concrete with a curing time of 30 days was investigated. Compared with the unconfined compressive strength of 30–0, the increase in 3SR-0 is 0.67 MPa, which is higher than the contribution value of 30% steel slag replacement ratio to the strength. This is caused by the fact that the silicon manganese slag had more pores, and the products formed after full contact with the cementing material filled between the structural gaps and promoted the internal compactness. When the cement dosage increased from 2% to 4%, the unconfined compressive strength of SR-60 increased by 2.80 MPa, which was much higher than the effect of waste slag, which was caused by the continuous participation of more CaO and other substances in the cement in the primary and secondary hydration.

### 3.3. Detection of Splitting Strength

The splitting strength is an important mechanical index of concrete, which can represent the tensile properties of concrete. Table 5 demonstrates the splitting values of concrete at different ages. It can be seen that the splitting strength is positively proportional to the curing time and cement content, and the difference laws of splitting strength were the same as that of unconfined compressive strength with the change in waste slag content, etc. The mechanism can also be explained by the regulation analyses of unconfined compressive strength. Compared with the concrete of pure reclaimed crushed stone aggregate, the splitting strength of 3SR-60 at 10 days increases by 0.23 MPa, with a growth rate of 54.8%, which once again showed that steel slag and silicon manganese slag can provide more minerals, and the internal structure of concrete was more complete through the hydration decomposition process.

### 3.4. Detection of Temperature Shrinkage

**Figure 10:**
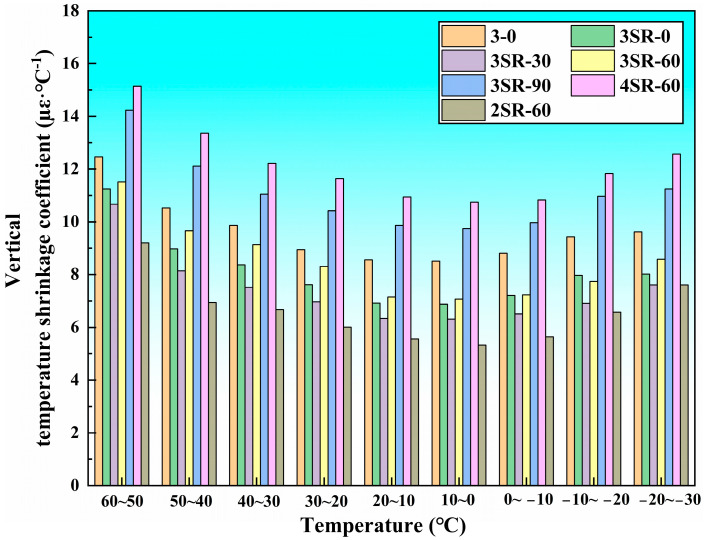
Test results of vertical temperature shrinkage coefficient.

**Figure 11:**
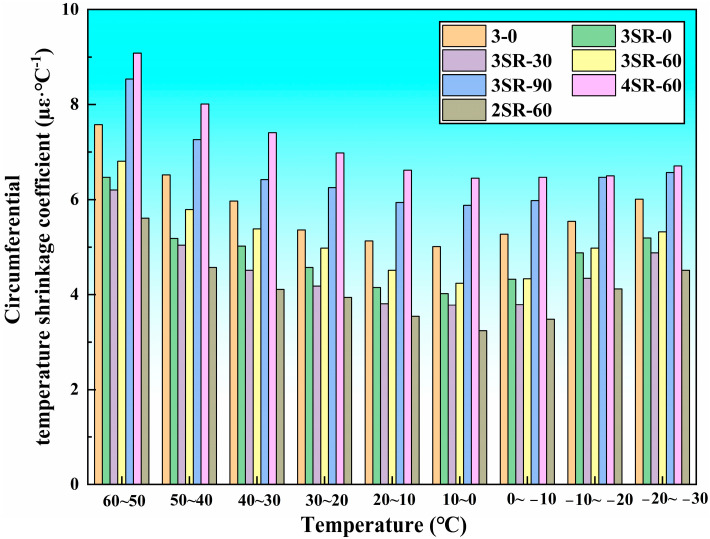
Test results of circumferential temperature shrinkage coefficient.

Figure 10 and Figure 11 summarize the relationship between vertical and circumferential temperature shrinkage coefficients and temperature specifications of 7 kinds of concrete. It can be seen that the change characteristics of temperature shrinkage coefficients of different specimens are basically the same, and the overall trend is decreasing and then increasing with the decrease in temperature. When the temperature range drops from 50–60 °C to 0–10 °C, the decrease rate of temperature shrinkage coefficient is large. Taking the vertical temperature shrinkage coefficient of 3SR-60 as an example, the decrease rate is 38.6%. When the temperature range continues to drop from 0–10 °C to −30–20 °C, the increase rate of the temperature shrinkage coefficient is relatively low. Taking the vertical temperature shrinkage coefficient of 3SR-60 as an example, the increase rate is 21.4%. The difference in amplitude between the two was caused by the difference in temperature sensitivity impacted by the different elastic modulus of multiple media in the mixture. In addition, the higher temperature range (0–60 °C) resulted in the shrinkage of the specimen volume, the interlocking aggregation of internal particles, the enhancement of compactness, and the improvement in temperature shrinkage resistance. In the range of 0–10 °C, the traction constraint of concrete reached the peak, and the movement potential was the weakest, resulting in the smallest temperature shrinkage coefficient. The vertical and circumferential temperature shrinkage coefficients are only 7.07 µε·°C^−1^ and 4.24 µε·°C^−1^, respectively. As the temperature continues to drop, the temperature shrinkage stress continues to expand, but the temperature shrinkage coefficient increases slowly due to its own adhesion. According to the research results and actual construction benefits, the construction temperature range should be controlled at 0–20 °C during the manufacture of road slopes.

Under all temperature specifications, the temperature shrinkage coefficients of 3–0, 3SR-0 and 3SR-30 decrease successively, while those of 3SR-30, 3SR-60 and 3SR-90 increase respectively. Taking the vertical temperature shrinkage coefficient at 50–60 °C as an example, the reduction values are 1.22 µε·°C^−1^ and 0.57 µε·°C^−1^. The upper value is 0.85 µε·°C^−1^, and 2.71 µε·°C^−1^, which was due to the continuous hydration of active mineral phases such as dicalcium silicate (C_2_S), tricalcium silicate (C_3_S), tricalcium aluminate (C_3_A) and tetralcium fluoroaluminate (C_4_AF) in steel slag and silicon-manganese slag, leading to increase in gelling yield and secondary mineral content in concrete, which have stronger temperature sensitive properties than gravel. The mechanical strength of 3–0, 3SR-0 and 3SR-30 is in a rising state. This is because compared with crushed stone, the high activity of silicon-manganese slag and the compactness of steel slag enhanced the temperature shrinkage resistance of the specimens, while the more gelling groups of 3SR-60 and 3SR-90 were dominated by the strong temperature excitation characteristics, and the temperature shrinkage coefficient became larger under the comprehensive influence. In addition, the strength of 3SR-90 was weaker than that of 3SR-60, and there were more hydration products, resulting in a decrease in its temperature shrinkage coefficient of 3.19 times that of 3SR-60.

When the cement dosage are 2% to 3% and 3% to 4%, the maximum vertical temperature shrinkage coefficient increase by 2.31 µε·°C^−1^ and 3.62 µε·°C^−1^, respectively. On the one hand, cement had a great influence on the temperature shrinkage deformation of the specimen, so the dosage of cement should be reasonably considered in actual engineering. On the other hand, the increment in temperature shrinkage coefficient increased with the increase in cement proportion, which was caused by the increase in hydration gelling.

### 3.5. Detection of Dry Shrinkage

After soaking the 7 groups of specimens for curing to 6 days successively for 24 h, the development trend of water content was detected, and the results are shown in Table 6.

As can be seen from Table 6, compared with 30-0, the moisture content of the specimen after the inclusion of silicon-manganese slag and steel slag of 4 specifications decreased by 8.9%, 16.5%, 23.9% and 26.8%, indicating that the moisture content of the specimen is negatively correlated with the proportion of waste slag. The main mechanism was that there are more active substances in silicon-manganese slag and steel slag, and the density of steel slag is higher than that of recycled macadam, resulting in better compactness of the specimen, and water did not easily to invade the specimen. Compared with 2SR-60, the moisture content of 3SR-60 and 4SR-60 decreases by 11.8% and 20.4%, respectively, indicating that the moisture content of specimens increases with the rise in cement dosage. The hydration degree is closely related to the proportion of cement. Micro particles of cement and C-S-H and calcium aluminate hydrate (C-A-H) produced by secondary hydration stuck together in the pores of the interface of silicon-manganese slag, steel slag, recycled macadam and fly ash transition zone, making the overall structure denser and reducing the interconnecting void between multiple media, resulting in the division of the original penetrating void into intermittent micro pores. It effectively hindered the water intrusion efficiency, so the moisture content of the specimen decreased with the increase in cement dosage after 24 h immersion.

**Figure 12:**
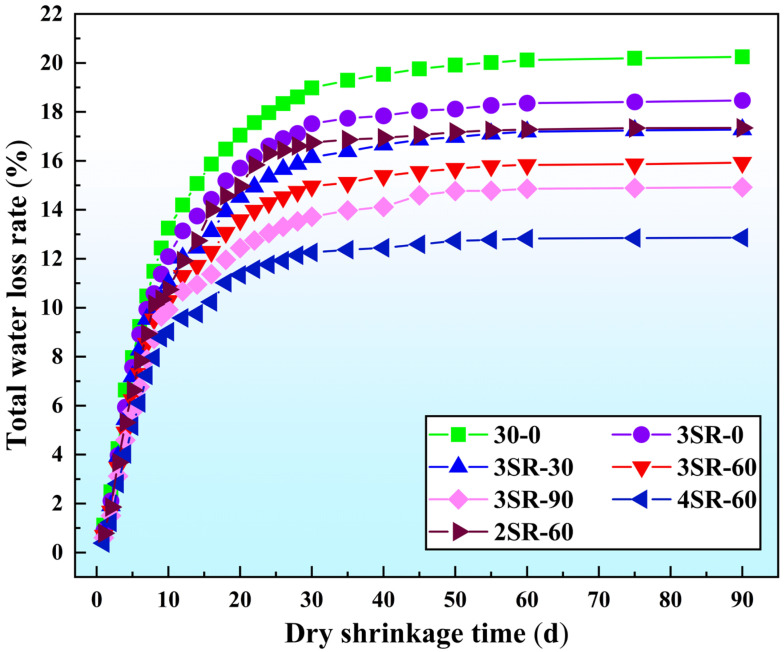
Relationship between total water loss rate and dry shrinkage age.

**Figure 13:**
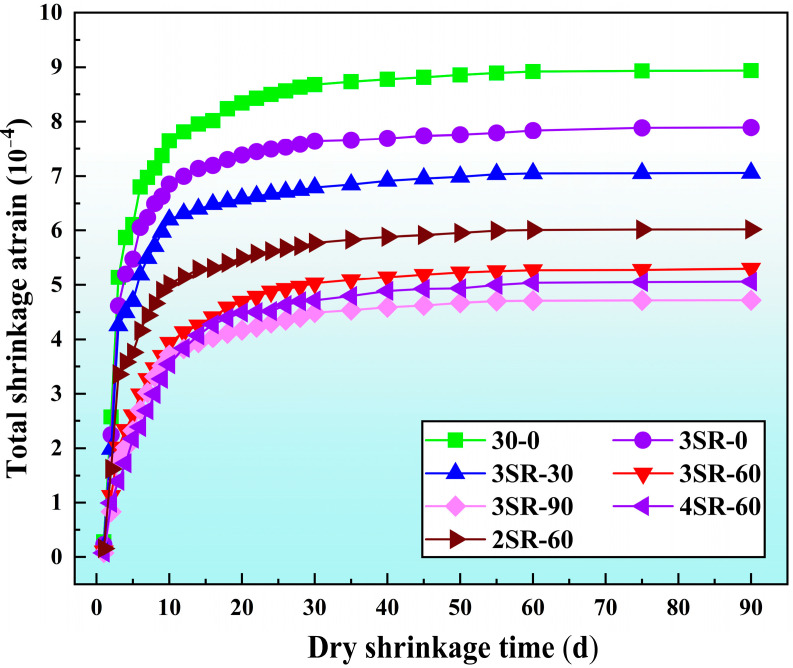
Relationship between total shrinkage strain and dry shrinkage age.

**Figure 14:**
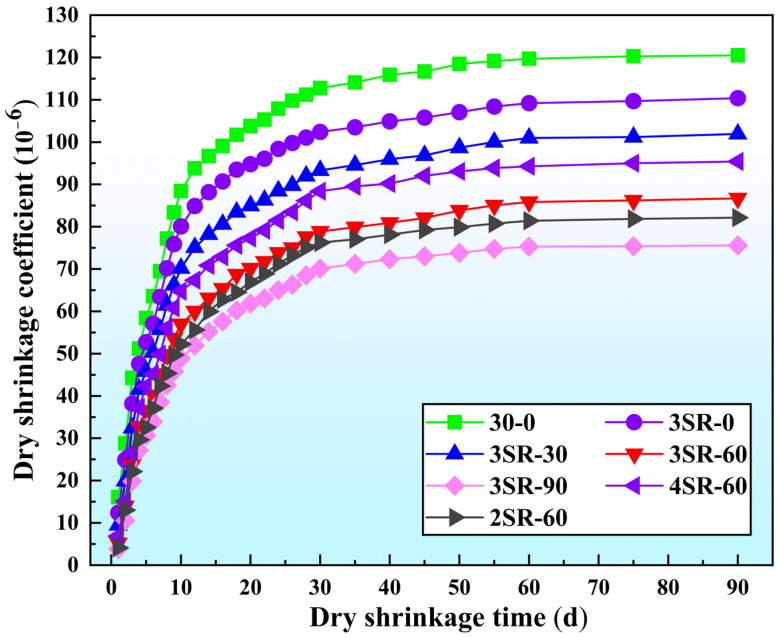
Relationship between total shrinkage coefficient and dry shrinkage age.

The dry shrinkage characteristics of 7 types of specimens were investigated in batches, and the development trends of total water loss rate, total dry shrinkage strain, dry shrinkage coefficient and dry shrinkage age were normalized. The results are shown in Figure 12, Figure 13 and Figure 14, respectively.

It can be seen from Figure 12 that the variation laws of the water loss rate of specimens with different media and dry shrinkage age are basically the same, indicating an increasing trend. At 90 d, the water loss rate of 30–0 to 3SR-90 decreases by 26.3%, indicating that the inclusion of waste slag effectively prevented the dissipation of water, which was caused by the better densification of steel slag and the worse water absorption rate than recycled macadam. When the cement dosage increases from 2% to 4%, the water loss rate of SR-60 at 90 days is 17.35%, 15.93% and 12.86%, indicating that the increase in cement proportion could effectively reduce the water loss rate of the mixture. When the drying time is 0–8 d, the slope of the curve is generally large, the water loss rate is fast, and the average water loss rate accounts for more than 60% of the total water loss rate. During 8–30 d of dry shrinkage, the mathematical curve gradually slows down and the growth degree of water loss decreases. The average water loss rate accounts for more than 90% of the total water loss rate. In the last 30 d, the slope of the curve tends to be constant. The rapid growth in the water loss rate in the early stages was caused by hydration consumption and partial evaporation of water, and the gradual slowing down in the middle and late stage was caused by dense products wrapped on the surface of aggregate particles to hinder hydration.

Figure 13 shows the same difference trend as the total water loss rate. This is because the dry shrinkage deformation of concrete depends on the water loss capacity to a large extent, and more water loss results in the narrowing of the spacing of internal particles, and the dry shrinkage strain increase [38]. At the age of 10 d, the dry shrinkage strain of 3SR-0, 3SR-30, 3SR-60 and 3SR-90 gradually decreases, the proportions are 10.4%, 18.9%, 48.4% and 51.5%, respectively, compared with that of only recycled macadam of aggregate specimen (30–0), which indicates that waste slag medium can enhance the dry shrinkage deformation resistance of a building materials system. On the one hand, the crushing value of steel slag is less than the crushed stone, which leads to the deformation of the particles in the specimens with a large proportion of steel slag. On the other hand, calcium hydroxide (Ca(OH)_2_) and magnesium hydroxide (Mg(OH)_2_) with micro-expansion characteristics were formed after hydration in the f-CaO and f-MgO in the steel slag and silicon-manganese slag, which increased the volume of the specimen and could compensate for part of the dry shrinkage deformation. It can be seen that when the waste slag was used to manufacture the drainage ditch in this study, its expansion ability could effectively control the dry shrinkage cracking defects of concrete. When the drying shrinkage time is 90 days, as the cement dose increases from 2% to 4%, the strain difference of SR-60 is 0.242 × 10^−4^ and 0.718 × 10^−4^ in sequence, indicating that the higher the cement dose, the more obvious the increase in strain value. The reason was that with the increase in cement content, the products such as C-S-H and C-A-H in the system increased, and the contraction source rose, which was highlighted as the dry shrinkage strain of the specimen increased. It can be seen that the proportion of waste slag and the dosage of cement in concrete are the main factors leading to the non-uniform of dry shrinkage deformation trend.

As can be seen from Figure 14, the drying shrinkage coefficient shows an upward trend with the deepening of the drying shrinkage process, and its growth law and generation mechanism are similar to the total drying shrinkage strain and the total drying shrinkage coefficient. With the increase in the amount of waste slag in concrete, the shrinkage coefficient decreases. The drying shrinkage coefficient increases with the increase in cement dosage. At 90 days, the drying shrinkage coefficients of 30–0, 3SR-0, 3SR-30, 3SR-60 and 3SR-90 are 120.5 × 10^−6^, 110.4 × 10^−6^, 101.9 × 10^−6^, 86.7 × 10^−6^ and 75.6 × 10^−6^, respectively, indicating that waste residue could significantly weaken the shrinkage capacity of the mixture. This is because the gradual dissipation of water in the specimen caused capillary shrinkage and thus dry shrinkage deformation, and the expansion of waste slag could compensate for this effect, and the higher the capacity of waste slag, the better the anti-dry shrinkage characteristics, reducing the reflection cracks of the specimen [39]. At this point, in practical application, an appropriate amount of industrial waste slag with expansion characteristics can be added, which can not only improve the dry shrinkage strain resistance of concrete, but also increase the utilization rate of waste slag, in line with the national green new development instructions.

### 3.6. Analysis of Micromorphology

**Figure 15:**
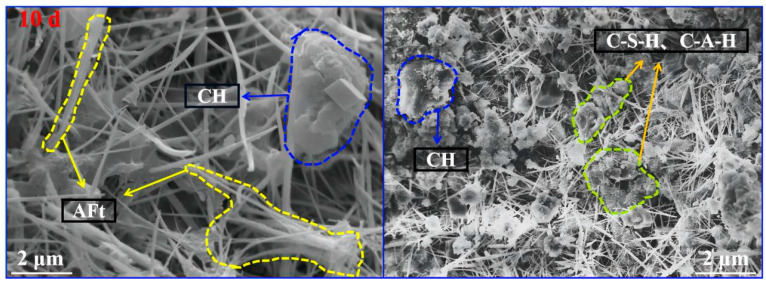

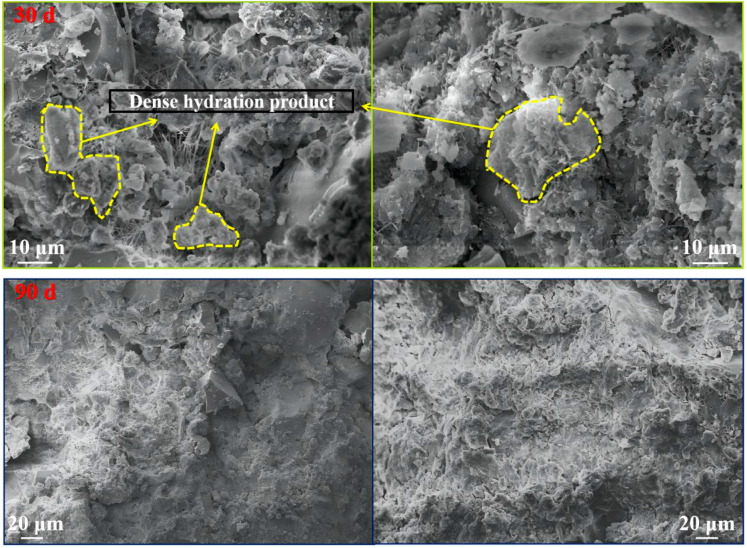
Micromorphology of the mixtures at different ages.

In order to comprehensively reveal the principle of difference caused by strength and dry shrinkage performance of specimens, the micro-morphology changes of the preferable specimen 3SR-60 at 10 d, 30 d and 90 d were respectively discussed, and the results are shown in Figure 15.

**Figure 16:**
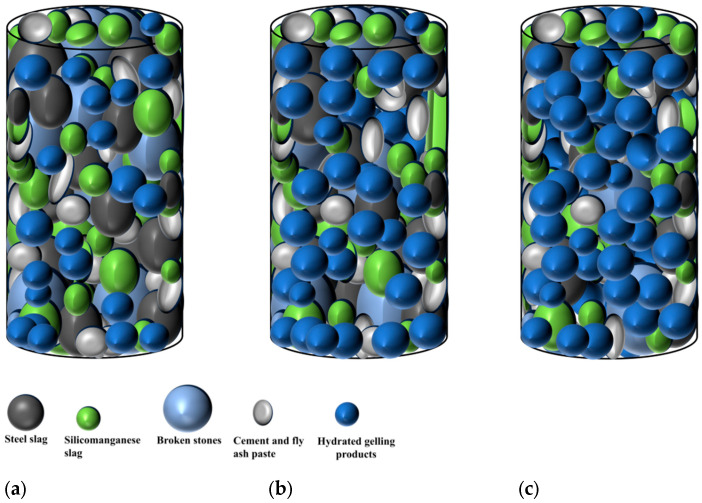
Microscopic change models at different ages of the specimen. (**a**) 7 d, (**b**) 28 d and (**c**) 90 d.

As shown in Figure 16, after drying for a duration of 10 days, a substantial presence of needle-bar hydrated gelling group (AFt), calcium hydroxide gelling (CH), and flocculent C-S-H and C-A-H is noticeable in the microstructure of the 3SR-60 specimen. These components exhibit an intricate interweaving within the structure. It was filled in the pores between aggregate particles and formed a spatial network system initially. In the early stage, hydration was more intense, and a large amount of water naturally dissipated, resulting in an increase in the number of pores inside the mixture and a larger pore size. In addition, the shrinkage deformation rate was faster due to the gradual increase in dry shrinkage stress. During 10 to 30 days of dry shrinkage, with the deepening of the hydration process, the overall spatial structure tended to be dense, and all products gradually developed and grew, and most of them demonstrated good dense gelling, which optimized strength of concrete gradually. This hindered the good contact between the media of the hydration system, resulting in a natural deceleration of the water loss rate and a reduction in the slope of the mathematical curve depicting dry shrinkage deformation over time. At 90 d, the microscopic morphology appeared to be the densest, and the mutual extrusion between the components made the overall structure have the strongest stability and the best resistance to external damage, and the interface structure was more perfect. The macroscopic results indicated that the variables of strength, total water loss rate, total dry shrinkage strain and dry shrinkage coefficient tended to be stable with the extension of time, which were consistent with the macroscopic variables investigated in the above mechanical and dry shrinkage tests.

Based on macroscopic detection results and microscopic mechanism analysis, this paper simulated the internal structural change model with high reproducibility as the hydration process deepens. The results are shown in Figure 16. It can be seen that with the extension of age, the gelling products increase and become dense and interspersed in the transition region between steel slag, silicon-manganese slag and gravel and the overall structural characteristics gradually transition from loose and high porosity to dense and polymerized, which is consistent with the results of the micro-morphology changes.

## 4. Discussion

In this paper, the impact of the waste slag mixing ratio, cement dosage and age on the mechanical strength and shrinkage of concrete has been investigated scientifically, but this is only an indoor study and has not been utilized in large-scale engineering. Therefore, in order to comprehensively evaluate the engineering adaptability of cement solid-waste concrete, the following topics need to be further carried out, as follows:

(1) The microscopic morphology of concrete at different ages was characterized by SEM, and the different mechanism of mechanical strength and dry shrinkage performance of concrete was revealed. Therefore, the subsequent operation should focus on observing the changing trend of pore size, quantity and shape with the deepening of curing, so as to fully reflect the microscopic characteristics of concrete;
(2) The unconfined compressive strength, splitting strength and shrinkage properties of concrete were discussed. Therefore, the evolution characteristics of concrete such as shear resistance and freeze resistance should be expanded;
(3) This paper also needs to analyze the economic and environmental benefits of cement solid-waste concrete and calculate the carbon reduction effect compared with traditional concrete.

## 5. Conclusions

Through scientific investigation of mechanical and contractile properties tests of 7 specimens with different proportions, the influences of waste slag replacement rate and cement dose on the mechanical and contractile properties of concrete with the increase in age were respectively discussed. The appearance of 3SR-60 at different ages was compared by SEM detection, so as to reveal the formation mechanism of the difference in resistance to external interference. The following conclusions are reached:

(1) The mechanical properties of concrete were directly proportional to the cement dosage and curing time. The unconfined compressive strength and splitting strength of 3SR-60 at 90 days reached 10.82 MPa and 1.02 MPa, which were higher than other types of concrete under the same dosage of cement;
(2) With the increase in cement dosage, the gelling of hydration products such as C-S-H, C-A-H and CH enhanced the sensitivity of the whole structure to temperature change, which led to the increase in temperature shrinkage coefficient. At the same time, the increase in the proportion of shrinkage source made the shrinkage stress inside the mixture gradually increase, and the shrinkage amount rose with the addition of cement dosage;
(3) With the incorporation of silicon-manganese slag and the increase in the steel slag substitution rate, the vertical and circumferential temperature shrinkage coefficients of the specimens were negatively correlated with the proportion of waste slag and then positively correlated, while the dry shrinkage coefficients decreased successively, which was caused by the difference in temperature shrinkage resistance and dry shrinkage ability of the multi-medium;
(4) The water loss of the specimen mainly occurred within 10 days of age, and the water loss rate could reach more than 60% of the total water loss rate. The dry shrinkage strain increased with the deepening of the dry shrinkage process, and the trend was first fast and then slow. At 60–90 days of age, the strain value tended to be constant. When the temperature decreased, the temperature shrinkage coefficient showed a decreasing trend and then an increasing trend, and the decreasing rate was larger above 0 °C, while the rising rate was slower below 0 °C;
(5) As the hydration process deepened, C-S-H, C-A-H, AFt and other gel groups continued to develop and interwove in the pores at the interface of the transition zone between composite aggregate and binder, making the microstructure denser and the integrity stronger. The macroscopic phenomenon demonstrated that the strength of concrete was stable, the water loss performance was reduced and the resistance to shrinkage was increased.

## Figures and Tables

**Table 1:** Chemical compositions of raw materials.

Materials	Mass Fraction(%)											
	CaO	Fe_2_O_3_	Al_2_O_3_	SiO_2_	K_2_O	MgO	Na_2_O	TiO_2_	SO_3_	MnO	P_2_O_5_	Trance Substances
Steel Slag	47.96	20.55	3.21	15.87	0.03	5.52	0.16	1.92	0.37	-	1.63w	2.78
Silicon-manganese slag	25.51	0.96	6.94	38.71	1.54	11.12	0.42	0.74	0.81	10.74	-	2.51
Fly ash	4.84	5.94	32.56	45.88	2.20	1.31	0.79	1.61	0.77	-	-	4.10
Cement	65.23	4.23	4.14	16.11	0.14	1.52	0.31	-	2.11	-	-	6.21
Recycled macadam	19.18	6.58	13.12	45.74	1.21	3.34	-	-	-	4.98	-	5.85

**Table 2:** Basic properties of aggregates.

Category	Particle Size (mm)	Crushing Value (%)	Water Absorption (%)	Apparent Density (g·cm^−3^)
Steel Slag	2.36–4.75	15.6	2.24	3.144
	4.75–9.5	16.4	2.01	3.142
	9.5–16	19.7	1.96	3.143
Silicon-manganese slag	0–2.36	/	8.69	1.986
Recycled macadam	0–2.36	/	4.88	2.656
	2.36–4.75	16.8		2.784
	4.75–9.5	18.4	4.15	2.895
	9.5–16	21.5	4.11	2.963
	16–26.5	21.9	3.96	2.984

**Table 3:** Mix ratios of concrete.

Number	Mass Ratio of Raw Materials in Concrete (%)						
	Silicon-Manganese Slag (mm)	Recycled Macadam (mm)	Steel Slag: Recycled Macadam (mm)		Recycled Macadam (mm)	Cement	Fly Ash
	0–2.36	0–2.36	2.36–9.5	9.5–16	16–26.5		
3–0	0	20.2	0:25.4	0:16.2	20.2	3	15
3SR-0	20.2	0	0:25.4	0:16.2	20.2	3	15
3SR-30	20.2	0	7.4:18.0	5.1:11.1	20.2	3	15
3SR-60	20.2	0	15.3:10.1	9.7:6.5	20.2	3	15
3SR-90	20.2	0	23.2:2.2	14.3:1.9	20.2	3	15
2SR-60	20.2	0	15.3:10.1	9.7:6.5	20.2	2	16
4SR-60	20.2	0	15.3:10.1	9.7:6.5	20.2	4	14

**Table 4:** The test results of optimum moisture content and maximum dry density.

Number	Optimum Moisture Content (%)	Maximum Dry Density (g/cm^3^)
3–0	10.4	1.846
3SR-0	11.2	2.077
3SR-30	10.8	2.194
3SR-60	10.3	2.317
3SR-90	9.8	2.429
2SR-60	10.0	2.277
4SR-60	10.7	2.426

**Table 5:** The test results of splitting strength at different ages.

Number	Splitting Strength at Different Ages (MPa)		
	10 d	30 d	90 d
3–0	0.42	0.61	0.68
3SR-0	0.51	0.73	0.82
3SR-30	0.56	0.77	0.89
3SR-60	0.65	0.91	1.02
3SR-90	0.61	0.85	0.94
2SR-60	0.54	0.76	0.83
4SR-60	0.77	1.01	1.13

**Table 6:** Moisture content of specimens immersed in water for 24 h.

Concrete Number	30-0	3SR-0	3SR-30	3SR-60	3SR-90	4SR-60	2SR-60
Moisture Content (%)	10.94	9.96	9.14	8.32	8.01	7.51	9.43

## Data Availability

The data supporting the findings of this study are available from the corresponding author upon reasonable request.

## Abbreviations

Full name	Abbreviation
calcium silicate hydrate.	C-S-H
ettringite	AFt
rice husk ash	RHA
Scanning Electron Microscope	SEM
dicalcium silicate	C_2_S
tricalcium silicate	C_3_S
tricalcium aluminate	C_3_A
tetralcium fluoroaluminate	C_4_AF
calcium oxide	f-CaO
magnesium oxide	f-MgO
